# Psychiatric and cognitive characterization of Jacobsen syndrome (Del11q)

**DOI:** 10.1192/j.eurpsy.2025.544

**Published:** 2025-08-26

**Authors:** A. Garriz-Luis, E. Rodríguez-Toscano, M. Burdeus-Olavarrieta, C. Arango, M. Parellada, N. Logo, C. M. Díaz-Caneja

**Affiliations:** 1 Department of Child and Adolescent Psychiatry, Institute of Psychiatry and Mental Health; 2 School of Psychology; 3 School of Medicine, Universidad Complutense, Madrid, Spain

## Abstract

**Introduction:**

Jacobsen Syndrome (JS), or 11q Deletion Syndrome, is a rare genetic disorder affecting approximately 1 in 100,000 births, with a female-to-male ratio of 2:1 (Grossfeld *et al.* Am J Med Genet A. 2004; 129A(1):51-61). It is caused by a deletion on chromosome 11’s long arm, leading to diverse clinical features, affecting mainly the immune and cardiac systems (Dalm *et al.* J Clin Immunol. 2015; 35(8):761-8). Core neuropsychiatric symptoms include intellectual disability, psychomotor delays and distinctive physical traits. Recent reports highlighted attention deficits and autism-like characteristics (Akshoomoff *et al.* Genet Med. 2015; 17(2):143-8). Although previous studies identified varied cognitive profiles in JS, most focused on medical features, and a detailed psychiatric and cognitive characterisation is lacking.

**Objectives:**

This study aims to describe and analyse the psychiatric and cognitive profiles of individuals with JS as well as its associations, within a Spanish sample.

**Methods:**

Twenty-nine participants aged 2 to 45 years were recruited from the Spanish association “11q España”. Psychiatric data were collected through interviews with parents and cross-referenced with medical reports, and behavioural symptoms were assessed using the Child Behavior Checklist (CBCL). Cognitive functioning was evaluated using Wechsler scales and the Merrill-Palmer-Revised scale.

**Results:**

The cohort’s average age was 12 years, with a female majority (68.9%). Psychiatric or neurodevelopmental comorbidities were present in over half (51.72%), with ADHD being the most common. According to the CBCL, 54.4% had internalising problems, and 54.5% had externalising problems. The average Intellectual Quotient (IQ) was 50.18, and the mean Developmental Quotient 36. When grouped by disability, 17.3% had borderline functioning, 17.3% mild, 21.7% moderate, 26% severe, and 17.3% profound ID. Higher IQ was associated with increased depression diagnoses and anxious/depressed symptoms.

**Conclusions:**

This study provides a detailed neurocognitive profile of individuals with JS, confirming its heterogeneous presentation. Psychiatric comorbidities, especially ADHD, were common, and cognitive functioning ranged from borderline to profound ID, with no cases of normal cognition. Notably, participants with higher cognitive abilities were more prone to depression, highlighting the need for targeted mental health support tailored to individuals with JS.

**Disclosure of Interest:**

None Declared

